# GOLM1 as a Potential Therapeutic Target Modulates B7-H3 Secretion to Drive Ovarian Cancer Metastasis

**DOI:** 10.1155/2022/5151065

**Published:** 2022-01-25

**Authors:** Junhua Guan, Yunpeng Qin, Guiyuan Deng, Haihong Zhao

**Affiliations:** ^1^Department of Obstetrics and Gynecology, The Fifth People's Hospital of Shanghai, Fudan University, Shanghai 200240, China; ^2^Anhui University of Chinese Medicine, Hefei, Anhui 230031, China; ^3^Teaching and Research Section of Clinical Nursing, Xiangya Hospital of Central South University, Changsha, Hunan 410000, China

## Abstract

**Introduction:**

This study was aimed at exploring whether the Golgi membrane protein 1 (GOLM1) enhanced ovarian cancer metastasis through B7-H3-dependent way.

**Methods:**

We collected the ovarian cancer patient samples from available databases including GEPIA, starBase, and Protein Altas that have GOLM1 and B7-H3 mRNA and protein expression. Ovarian cancer cell line SKOV3 was purchased. Knockdown GOLM1 and B7-H3 cell lines were obtained through introducing shRNAs by lentivirus package system, while GOLM1 or B7-H3 overexpression cell line was obtained by introducing GOLM1 full-length gene. Furthermore, wound-healing assay and Transwell assay were performed to assess tumor invasion and metastasis abilities; related proteins' expression was quantitated by western blotting, ELISA, and flow cytometry assay. The protein interaction was quantified by co-immunoprecipitation.

**Results:**

GOLM1 has the correlative expression pattern with B7-H3 in ovarian cancer through patient sample databases (*R* = 0.421). GOLM1 knockdown had minimal impact on B7-H3 mRNA synthesis, while downregulated B7-H3 protein expression on tumor membrane and soluble B7-H3 (sB7-H3) level (*p* < 0.05) through physical interaction, GOLM1 knockdown, significantly reduce tumor invasion and metastasis in vitro (*p* < 0.05). Moreover, exogenous sB7-H3 significantly rescued this inhibitory effect. Both GOLM1 and B7-H3 knockdown restrained tumor growth and metastasis in immunodeficient mice and prolonged the survival rate.

**Conclusions:**

GOLM1 acts as an initial oncogenic driving gene by promoting ovarian cancer invasion and metastasis through modulating B7-H3 protein maturation and secretion.

## 1. Introduction

Ovarian cancer (OV) is the second leading cause of gynecologic cancer death in women around the world. The disease is often diagnosed at late stage; thereby, the outcome is quite unpleasant [1]. Over the past decade, minimal improvement in mortality control has been achieved. It was widely accepted that metastasis is mostly responsible for the high mortality and morbidity in women with OV. Unfortunately, the mechanisms behind this process are still not fully understood [2]. Therefore, it is urgent to survey the potential therapeutic target for this drastic malignant tumor.

Compared with traditional therapy, chimeric antigen receptor (CAR) T cells are an innovative and effective approach in fighting against cancers [3]. Our previous study has proved that redirected B7-H3 CAR-T cells can effectivity eliminate anaplastic lymphoma kinase-positive anaplastic large-cell lymphoma [4]. Moreover, recent studies have found that redirected B7-H3 CAR-T cells effectively control ovarian cancer without obvious toxicity. To better enhance this B7-H3 targeting therapy, the mechanism of B7-H3 regulation needs to be thoroughly elucidated [5]. B7-H3, which belongs to the B7 immunoglobulin superfamily, was initially discovered as a T cell-stimulating protein [6], but subsequent studies found that B7-H3 inhibited T cell cytotoxicity that permitted tumor proliferation and metastasis [7, 8]. Despite its immunoregulatory role, B7-H3 is also emerging as an oncogene that regulates tumor growth, metastasis, and drug sensitivity [9]. B7-H3 was overexpressed in ovarian cancer and enhanced tumor cell invasion and migration through activating Jak2-STAT3 pathway [10]. Moreover, soluble B7-H3 form (sB7-H3) was also excessively found in neoplastic blood vessels, regulating VEGF expression in tumor cells [11], which suggested that B7-H3 plays an important role in ovarian tumor angiogenesis and metastasis. Even through the majority of normal human tissues had B7-H3 mRNA expression, B7-H3 protein expression was highly conserved through tight posttranscriptional regulation in human cells [12].

As a Golgi membrane protein, GOLM1 was related to cargo transport process to license proteins into subsequent subcellular location [13]. GOLM1 has recently been proved as a key player of hepatocellular carcinoma metastasis, acting as an adaptor for spatial redistribution and recycling of epidermal growth factor receptor. GOLM1 expression has positive correlation with early recurrence, metastasis, and poor survival outcome [14]. Moreover, GOLM1 is highly expressed in the serum of several types of tumors, such as breast, prostate, lung, cervical cancer, etc. There are a growing number of studies that show GOLM1 as one of the most promising markers for early diagnosis and prognosis of those cancers [15–18]. However, GOLM1 expression pattern in ovarian cancer was not well studied yet.

In the current study, we found that GOLM1 had correlative expression pattern with B7-H3 in ovarian cancer database. We demonstrated that GOLM1 was the key regulator of B7-H3 protein formation and secretion; reduced GOLM1 levels dampened B7-H3 secretion, which is initial for tumor metastasis and invasion both in vitro and in vivo. Both of these evidences suggest that GOLM1 was important for ovarian cancer development through regulating B7-H3 protein distribution, which provide further insight towards the B7-H3 targeting therapy and mechanism for ovarian cancer.

## 2. Materials and Methods

### 2.1. Bioinformatics Analysis

The correlation of B7-H3 and GOLM1 transcriptional level was analyzed and plotted on starBase (http://starbase.sysu.edu.cn/index.php); protein correlation was analyzed and plotted on Protein Atlas database (https://www.proteinatlas.org). The survival plot was analyzed on KM plotter (https://kmplot.com).

### 2.2. Sequence, Plasmid, and Transfection Reagents

Three GOLM1 shRNA targets are #1 : 5′-GTTGAGAAAGAGGAAACCAAT-3'; #2 : 5′-GAACAGTGTGAGGAGCGAATA-3'; and #3 : 5′-CGAATAGAAGAGGTCACCAAA-3'. B7-H3 shRNA sequence is 5′-GGACAAGAAATTGCTTGATTG-3′; these shRNA nucleotides were synthesized by Genewiz. Lentiviral vectors pLKO.1, psPAX2, and pMD2.G were purchased from BioFeng. The GOLM1 full-length coding sequence was cloned from SKOV3 genome into pcDNA3.1 vector. pCDH-EF1-Luc-T2A-tdTomato was purchased from Youbio for bioluminescence imaging in vivo.

PEIpro was used for transfection and lentivirus package according to the manufacturer's instructions (Polyplus).

### 2.3. Cell Culture and Treatment

SKOV3 cell lines was cultured in Micro-5A medium (Gibco). Letivirus package 293T was cultured in DMEM medium (Gibco), with 10% FBS (Gibco) and 100 U/ml penicillin/streptomycin. Cells were incubated at 37°C with a 5% CO2 atmosphere. For in vitro metastasis assay, the cells were treated with 10 *μ*g/ml soluble human B7-H3 (R&D) for indicated times.

### 2.4. Quantitative Real-Time PCR (qPCR)

TRIzol reagent was used for total RNA extraction, first-strand cDNA was synthesized according to the manufacturer's protocol (Yeasen), and real-time PCR was performed on CFX qPCR instrument (Bio-Rad). B7-H3 primers are F: 5′-GTGGGGCTGTCTGTCTGTCTCAT-3′, R: 5′-GCTGTCAGAGTGTTTCAGAGGCT-3′; GOLM1 primers: F: 5′-AGAGCGTCAACAAGCTGTACC-3′, R: 5′-CAGAGGAATTACGGCAGGCTG-3′; actin primers: F: 5′-AGCACA- GAGCCTCGCCTTT-3′, R: 5′-AGAGGCGTACAGGGATAGCA-3′.

### 2.5. Western Blotting

Cells (1 × 10^6^) were collected and washed with PBS three times. The total protein was extracted by lysis buffer (Beyotime) containing 1X proteinase inhibitor (Roche) for 30 minutes on ice. The supernatants were collected by centrifugation. 20 *μ*g of protein was loaded on SDS-PAGE (12.5%) and transferred onto the PVDF membranes. After being blocked by notfat milk, diluted primary antibodies and relevant HRP conjugated secondary antibodies were incubated, ECL detection reagents and X-ray film espousing were used to detect the protein expression. The GOLM1, B7-H3 antibody (Abcam), actin, VEGF, MMP-9 antibody (Santa Cruz), and E-cadherin antibody (CST) were used according to the manufacturer's recommendations.

### 2.6. Flow Cytometry

Cells (1 *∗* 106 cells/ml) were incubated with the rabbit mAb against human B7-H3 (Abcam) for 30 min at 4°. Then, FITC conjugated donkey-anti-rabbit Fc mAb (Ebioscience) was added to the cells, incubated for another 20 min at 4°. The expression of membrane B7-H3 by the cells was analyzed by flow cytometry (BD, Accuri C6).

### 2.7. ELISA

ELISA kit (R&D) was used to measure the levels of soluble B7-H3 in the supernatants and mice blood. Briefly, the mice blood was obtained from the orbit and centrifuged for 20 min at 1000 g. Cultured cell supernatants were centrifuged for 20 min at 800 g. The samples were transferred to the plate. Incubated for 2 h at 37°C, the supernatants were thoroughly removed, and conjugate and substrate solution were added into each well. Stop reaction by adding stop solution; read at 450/540 nm using Multiscan Spectrum (Molecular Devices).

### 2.8. Co-Immunoprecipitation

Cells (at least 1.5 × 10^7^) were harvested and lysed within ice-cold RIPA (150 mM NaCl, 50 mM Tris pH7.6, with 1 mM PMSF, 1 g/ml leupeptin, 1 g/ml aprotinin, and 1 g/ml pepstatin) for 30 min. The lysates were centrifuged at 12,000 g for 5 min at 4°C to remove cell debris. Five percent of the supernatant was used as input. The remaining supernatant was then incubated with the indicated primary antibody or IgG at 4°C with rotation overnight. Next day, the supernatants were incubated with 35 mL protein A/G Sepharose beads at 4°C with rotation for another 3 hours. For immunoblotting assays, the immunoprecipitates were boiled in 6x SDS loading buffer. The protein was separated by SDS-PAGE and transferred to nitrocellulose membrane for immunoblotting with primary antibodies or IgG and appropriate secondary antibodies. The membrane was scanned with an Odyssey western blot scanner (LI-COR Biosciences).

### 2.9. Transwell Migration Assay

Cells (5 *∗* 104) with or without soluble B7-H3 in 200 *μ*l Micro-5A medium were seeded into the Matrigel-coated (BD) upper chambers, and 500 *μ*l of Micro-5A containing 10% FBS was added into the lower chamber. The chamber was settled at 37°C with 5% CO2 for 48 hours then washed with PBS; the cells were fixed and stained with crystal violet dye. The invaded number of cells was pictured in 6 random fields under microscope (Olympus) and analyzed by ImageJ software.

### 2.10. Wound-Healing Assay

Cells (1 *∗* 104) with or without soluble B7-H3 were seeded into 6-well plates and incubated overnight. When the cells reached 95% confluence, cell monolayers were scraped with a 10 *μ*l pipet tip, and photographs of four regions per scratch were taken at 0 and 48 hours under microscope (Olympus). Triplicate independent experiments were performed, and representative images were analyzed using ImageJ software.

### 2.11. Mice Experiments

The mice study was conducted in accordance with the recommendations of the National Institutes of Health guide for the care and use of Laboratory animals (NIH Publications no. 8023, revised 1978). All experimental protocols were approved by the Fudan University Animal Care Committee. NOD/SCID mice (6 weeks old, female) were obtained from Vital River Laboratory. For subcutaneous tumor formation model, 1 *∗* 10^7^ luciferase stably expressed SKOV3 cells in 100 *μ*L PBS were injected subcutaneously into the mice. Mice were monitored both at day 0 and day 42 after injection for tumor growth by bioluminescence imaging with the Xenogen Spectrum System and Living Image software version 3.2 (Caliper, PerkinElmer), For tumor metastasis model, 1 ∗ 10^7^ cells were injected through tail vein and imaging at day 0 and day 42.

### 2.12. Statistical Analysis

The statistical data were analyzed and depicted by GraphPad Prism version 8.2. All data are shown as means with standard error of mean (SEM). Unpaired *t*-test was used to display significant difference. *p* value difference was calculated to show statistical significance (^*∗*^*p* < 0.05,  ^*∗∗*^*p* < 0.01,  ^*∗∗∗*^*p* < 0.001).

## 3. Results

### 3.1. GOLM1 Is Correlated with B7-H3 Expression in Human Ovarian Cancer

**Figure 1:**
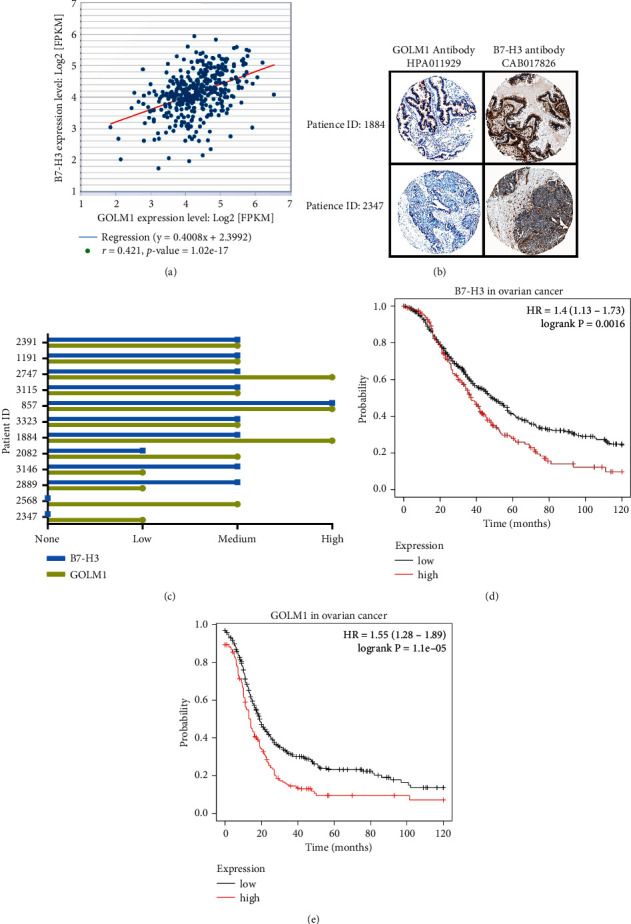
GOLM1 is correlated with B7-H3 expression in human ovarian cancer. (a) The mRNA expression of GOLM1 and B7-H3 was analyzed with the Pearson Correlation analysis GEPIA database |Log2FC| cutoff = 1; *q*-value cutoff = 0.01; ANOVA differential analysis in 426 ovarian cancer (OV) samples and 88 normal ovarian tissue. (b) The GOLM1 and B7-H3 mRNA levels were retrieved from OV dataset (*n* = 379) with ENCORI database, *R* = 0.421. (c) IHC plot of the typical protein expression of GOLM1 and B7-H3 in two OV patients (ID: 1884 and 2347) from The Human Protein Atlas. (d) Statistical analysis of GOLM1 and B7-H3 protein expression in 12 patients from patients with OV from The Human Protein Atlas. (e) Kaplan–Meier plot of the overall survival of 1402 ovarian cancer patients in TCGA database. The logrank *p* value was 0.0016.

In order to detect the B7-H3 and GOLM1 expression pattern in ovarian cancer, we searched the starBase v3.0 project [19] to directly show the correlation of GOLM1 and B7-H3 transcription level in 379 ovarian cancer samples. The regression analysis showed that the transcriptional level of GOLM1 was positively related to B7-H3 in clinical pathology (Figure 1(a); *r* = 0.421, *p* value = 1.02−17). Next, we collected 12 ovarian cancer patients from Human Protein Atlas [20]. Consistently, we found that ovarian cancers with high B7-H3 expression also had comparable GOLM1 expression in protein level, while tumor with less B7-H3 expression had barely GOLM1 expression (Figure 1(b)). A total of 12 patient samples indicated that B7-H3 highly expressed tumors tend to have GOLM1 overexpression (Figure 1(c)). Furthermore, we analyzed the prognostic significance of B7-H3 and GOLM1 in ovarian cancer, which showed that high B7-H3 expression correlated with shorter overall survival (median survival = 37 months vs. 48 months) than patients with low B7-H3 expression (Figure 1(d)), while high GOLM1 expression also correlated with shorter overall survival (median survival = 13.73 months vs. 19 months) than patients with low B7-H3 expression (Figure 1(e)). Collectively, these results indicated that B7-H3 was positively correlated with GOLM1 expression and predicted poor prognosis in patients with ovarian cancer.

### 3.2. B7-H3 mRNA Expression Is Partially Regulated by GOLM1

**Figure 2:**
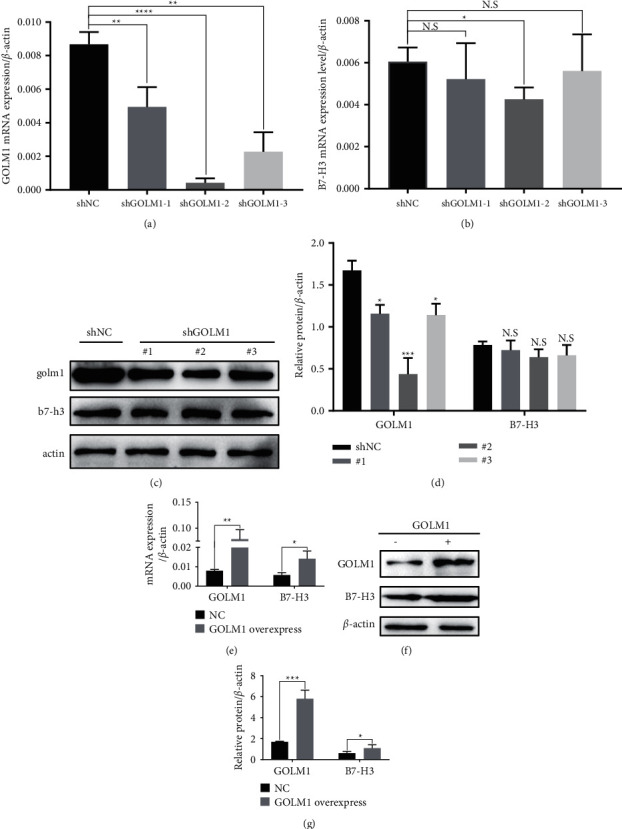
B7-H3 expression is partially regulated by GOLM1. (a) qPCR analysis of GOLM1 knockdown efficiency in SKOV3 cell line after stable introduction of three different shRNAs by lentivirus system. (b) qPCR analysis of B7-H3 mRNA expression in three GOLM1 shRNA stable induced SKOV3 cell lines. (c) Western blot analysis of GOLM1 and B7-H3 protein expression in three GOLM1 knockdown SKOV3 cell lines. (d) Statistics of GOLM1 and B7-H3 protein expression in three GOLM1 knockdown SKOV3 cell lines. (e) qPCR analysis of GOLM1 and B7-H3 expression in SKOV3 cell line with transient exogenous expressed GOLM1 using PEI proreagent. (f) Western blot analysis of GOLM1 and B7-H3 expression in GOLM1 overexpressed SKOV3 cell line. (g) Statistics of GOLM1 and B7-H3 expression in GOLM1 overexpressed SKOV3 cell line. Each experiment was repeated three times and values are presented as means ± SEM (^*∗*^*p* < 0.05,  ^*∗∗*^*p* < 0.01,  ^*∗∗∗*^*p* < 0.001, two-tailed Student's *t*-test).

To study whether GOLM1 regulated the expression of B7-H3, three GOLM1 knockdown shRNA sequences were used for lentivirus package with 3rd package system in 293T cells. Next, we infected the SKOV3 cell line with these three lentiviruses independently. The GOLM1 knockdown efficiency was detected by qPCR (Figure 2(a)); #1 and #3 shRNA reduced 2- or 3-fold of GOLM1 expression, while #2 shRNA has 10-fold of GOLM1 knockdown efficiency. Then, we used qPCR and Western blot to detect the B7-H3 mRNA and protein expression levels in these three cell lines (Figures 2(b) and 2(c)); only #2 shRNA had tiny downregulated effect of the B7-H3 transcription level. Interestingly, three shRNAs did not affect the B7-H3 total protein level, and three independent results were calculated in Figure 2(d). Next, we transiently induced exogenous GOLM1 expression plasmid into SKOV3 cell lines. Surprisingly, we found that exogenous GOLM1 expression increased B7-H3 mRNA expression about 3-fold (Figure 2(e)). Accordingly, GOLM1 overexpression enhanced B7-H3 protein level about 5-fold (Figures 2(f) and 2(g)). These results indicated that GOLM1 may not be the direct regulator of B7-H3 mRNA synthesis, while enhanced GOLM1 indeed regulated B7-H3 protein level.

### 3.3. GOLM1 Expression Was Correlated with B7-H3 Membrane Protein Expression and B7-H3 Secretion through Physical Interaction

**Figure 3:**
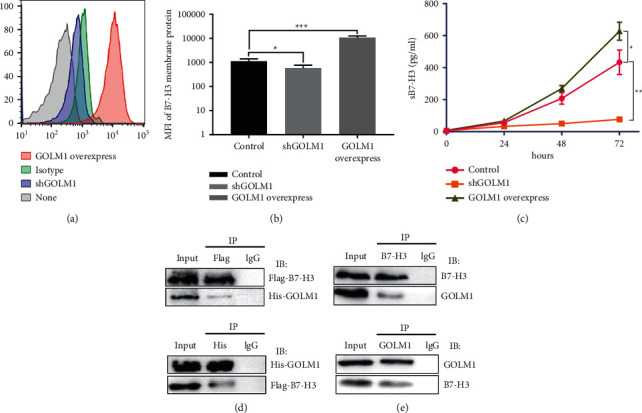
GOLM1 expression was correlated with B7-H3 membrane protein expression and B7-H3 secretion. (a) FACS analysis of comparable B7-H3 membrane expression in GOLM1 knockdown and GOLM1 overexpressed SKOV3 cell lines. Original SKOV3 cell line as the control. (b) Statistics of B7-H3 membrane expression in three SKOV3 cell lines indicated in (a). (c) ELISA assay of soluble B7-H3 expression in three SKOV3 cell lines after cultured in incubator for three indicated times (24 hours, 48 hours, and 72 hours). Three independent experiments are performed, and values are presented as means ± SEM (^*∗*^*p* < 0.05,  ^*∗∗*^*p* < 0.01,  ^*∗∗∗*^*p* < 0.001, two-tailed Student's *t*-test). (d) The interaction of exogenous expressed GOLM1 and B7-H3 was detected in 293T cells cotransfected with His-GOLM1 and Flag-B7-H3. (e) The interaction endogenously expressed GOLM1, and B7-H3 was detected in SKOV3 cells.

GOLM1 was identified independently in the “Discovery of Secretory Protein Program,” which controls multiple protein maturation and distribution [21]. Upregulated GOLM1 may assist in B7-H3 maturation and augment B7-H3 flux intratumor cells, whose in turn feedback regulated B7-H3 mRNA synthesis. Thereby, we used flow cytometry to detect B7-H3 expression on cell membrane. Surprisingly, GOLM1 knockdown significantly reduced B7-H3 capacity, while GOLM1 overexpression significantly enhanced B7-H3 capacity on cell membrane (Figure 3(a)). Statistical analysis of mean fluorescence intensity (MFI) of B7-H3 membrane protein was shown in Figure 3(b) (^*∗*^*p* < 0.05,  ^*∗∗∗*^*p* < 0.001). Moreover, we also detected soluble B7-H3 in cultured medium; compared with original SKOV3 cell lines, GOLM1 knockdown retained soluble B7-H3 in an extremely low level, while GOLM1 overexpression significantly enhanced soluble B7-H3 in the medium (Figure 3(c)). Collectively, we proved that GOLM1 was associated with B7-H3 maturation and enhanced soluble B7-H3 form out of tumor cells. To elucidate the mechanisms underlying how GOLM1 increases B7-H3 expression, the plasmids encoding His-tagged GOLM1 and FLAG-tagged B7-H3 were cotransfected into 293T cells, followed by the co-immunoprecipitation (co-IP) assay. The interaction of GOLM1 and B7-H3 was visualized by the co-IP assay and western blotting, and the reciprocal co-IP assay confirmed that GOLM1 could bind to B7-H3 (Figure 3(d)). To ensure that the interaction we observed also occurs in endogenous level of ovarian cancer cells, we performed co-immunoprecipitation experiments with lysates isolated from SKOV3 cells. Immunoprecipitation with anti-GOLM1 antibody followed by western blotting with anti-B7-H3 antibody and vice versa indicated that GOLM1 and B7-H3 physically interacted in SKOV3 cells (Figure 3(e)).

### 3.4. GOLM1 Regulates Ovarian Cancer Metastasis and Invasion Ability in Soluble B7-H3-Dependent Manner

**Figure 4:**
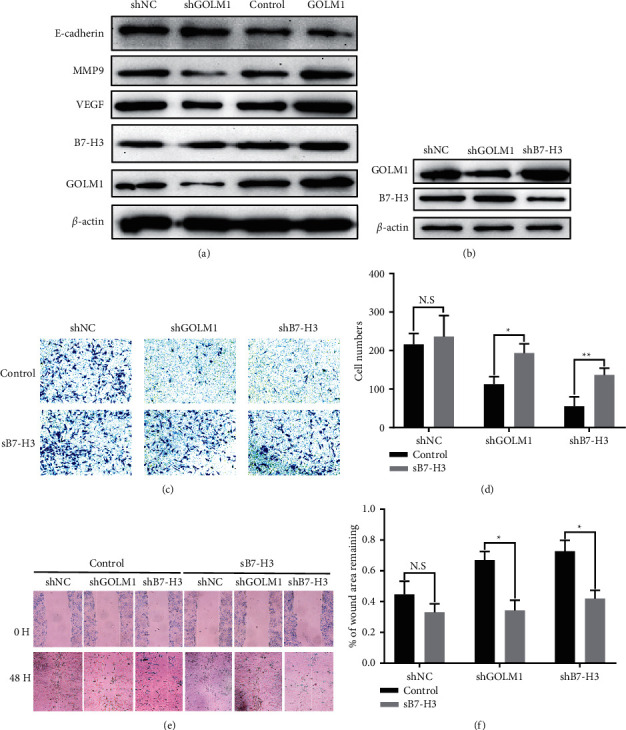
GOLM1 regulates ovarian cancer metastasis and invasion ability in soluble B7-H3-dependent manner. (a) Western blot of B7-H3, GOLM1 and metastasis-related protein E-cadherin, MMP-9, and VEGF in GOLM1 knockdown and GOLM1 overexpressed SKOV3 cell lines. Both scramble shRNA knockdown SKOV3 cell line and original SKOV3 cell line are treated as the control. (b) Western blot of lentivirus induced stable knockdown B7-H3 in SKOV3 cell lines compared with original and GOLM1 knockdown SKOV3 cell lines. (c) The Transwell assay to determine the invasiveness of GOLM1 knockdown and B7-H3 knockdown SKOV3 cell lines treated with or without additional soluble B7-H3 (10 *μ*g/ml) for 48 hours. (d) Statistics of the invaded cell number between each group (^*∗*^*p* < 0.05,  ^*∗∗*^*p* < 0.01). (e) The wound-healing ability of GOLM1 knockdown and B7-H3 knockdown SKOV3 cell lines treated with or without soluble B7-H3 (10 *μ*g/ml) for 48 hours. (f) Statistics of the percentage of wound area remaining between each group (^*∗*^*p* < 0.05).

Soluble B7-H3 had been confirmed to facilitate metastasis through increasing the formation of premetastatic niches through regulating the expression of VEGF in tumor cells [11], while VEGF induced the expression of MMP-9 was crucial for the metastasis of ovarian cancer cells [22]. Here, we found that GOLM1 knockdown significantly reduced VEGF and MMP-9 expression compared with scramble knockdown group; on the contrary, GOLM1 overexpression significantly upregulated VEGF and MMP-9 expression in ovarian cancer cells. E-cadherin, the marker of epithelial-mesenchymal transition, was also negatively related with GOLM1 expression (Figure 4(a)). This gave us a cue that GOLM1 regulating ovarian cancer cell metastasis was dependent on the soluble B7-H3. To testify this hypothesis, we constructed the B7-H3 stable knockdown SKOV3 cell line as the positive control. The knockdown efficiency was detected by western blot. Meanwhile, GOLM1 expression was not changed with B7-H3 knockdown (Figure 4(b)). Next, we evaluated cancer cell migration by using in vitro Transwell assay and the wound scratch assay. In the normal culture condition, we found that both GOLM1 knockdown and B7-H3 knockdown significantly reduced invasive cell numbers compared with control group. While with additional soluble B7-H3 condition, the invasive ability was restored in both GOLM1 and B7-H3 knockdown group (Figures 4(c) and 4(d)). Accordingly, soluble B7-H3 also rescued the impaired wound closing ability in GOLM1 and B7-H3 knockdown group (Figures 4(e) and 4(f)). Above all, these results suggest that GOLM1 can increase ovarian cancer cell invasion and migration in vitro by upregulated soluble B7-H3 expression.

### 3.5. GOLM1 Regulates Ovarian Cancer Progression In Vivo

**Figure 5:**
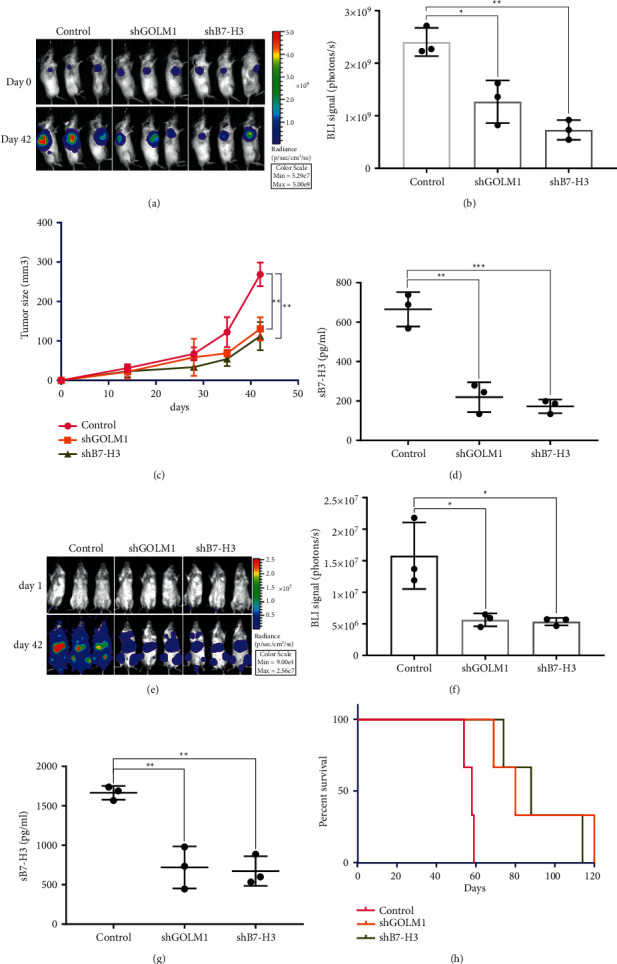
GOLM1 regulates ovarian cancer progression in vivo. (a) In vivo bioluminescence imaging of 107 GOLM1 and B7-H3 knockdown SKOV3 cell lines subcutaneous injected behind the right upper limb in immunodeficient NSG mice, using original SKOV3 as the control. Each group contains three mice. The imaging was performed at day 0 and day 42. (b) Statistics of BLI signal between three groups (^*∗*^*p* < 0.05,  ^*∗∗*^*p* < 0.01). (c) Vernier caliper measurement of tumor volume every 7 days after tumor inoculation. Statistical difference was calculated at day 42 (^*∗*^*p* < 0.05,  ^*∗∗*^*p* < 0.011). (d) ELISA assay of soluble B7-H3 in peripheral blood of each mouse at day 42 (^*∗∗*^*p* < 0.01,  ^*∗∗∗*^*p* < 0.001). (e) In vivo bioluminescence imaging of 107 GOLM1 and B7-H3 knockdown SKOV3 cell lines' tail vein injected in immunodeficient NSG mice, using original SKOV3 as the control. Each group contains three mice. The imaging was performed at day 1 and day 42. (f) Statistics of BLI signal between the three groups (^*∗*^*p* < 0.05). (g) ELISA assay of soluble B7-H3 in peripheral blood of each mice at day 42 (^*∗∗*^*p* < 0.01). (h) Survival curve of the mice in the three groups.

To assess the effects of GOLM1 on tumor growth in vivo, we generated SKOV3 cell lines stably expressing firefly luciferase with GOLM1 knockdown or B7-H3 knockdown. The original SKOV3 cells formed obvious large tumors after 6 weeks of subcutaneous injection into SCID mice through bioluminescence imaging (Figures 5(a) and 5(b)), while mice injected with SKOV3 GOLM1 knockdown cells developed relatively smaller tumors. The tumor growth curves were also measured by vernier caliper, which also indicated the same results (Figure 5(c)). At day 42, we used ELISA assay to test the soluble B7-H3 in mice blood; accordingly, GOLM1 knockdown group had a significant low level of sB7-H3 similar with B7-H3 knockdown group (Figure 5(d)). To assess the effects of GOLM1 on tumor metastasis, we separately injected the above cell lines through tail vein to monitor the bioluminescence signal after 6 weeks (Figure 5(e) and 5(f)). We found the GOLM1 knockdown cells developed quite weak signal compared with control group, which indicate less metastasis sites were formed in this group. And blood test of sB7-H3 level in GOLM1 knockdown group was also significantly reduced (Figure 5(g)). Moreover, the survival time of GOLM1 knockdown group is obviously longer due to the reduced metastasis sites formation. Collectively, we proved that GOLM1 regulates ovarian cancer progression and metastasis though enhancing soluble B7-H3 level.

## 4. Discussion

Ovarian cancer (OV) is an aggressive malignancy that is often refractory to standard treatments. In recent years, no favorable advances have been found in the treatment of OV; debulking surgery followed by taxane/platinum-based chemotherapy is still the standard of care. Unfortunately, about 90% of patients with advanced-stage ovarian cancer will experience recurrence that is generally incurable [23]. Although current immunotherapies have shown strong potency for human cancer, the efficacy of PD-L1 or PD-1 blockade in OV has been worse than other tumor types—only 11.5–12.3% ORR in PD-L1-positive OV patients while 5.9% ORR in PD-L1-negative OV patients [24]. Instead, another checkpoint molecule, B7-H3, was highly expressed by both OV tumor cells and tumor-associated epithelial cells, which suggest that B7-H3 may be a potential target, particularly in OV patients [25]. Indeed, targeting B7-H3 via chimeric antigen receptor T cells has strong antitumor responses in the absence of toxicity in OV tumors [5]. Despite its strong antitumor potency, the regulatory mechanism of B7-H3 expression is still not clear. Further understanding of this mechanism could be helpful for forward improvement of anti-B7-H3 treatments for OV patients.

B7-H3 protein expression in normal human tissues is strictly regulated. While in many types of malignant tumors, B7-H3 is commonly overexpressed and elevated in an extracellular form, suggesting that tumor cells obtain the ability to break the B7-H3 posttranscriptional regulation. Here, we first demonstrated that as a metastasis-related prooncogene, GOLM1 is correlated with B7-H3 expression both on transcription level and protein level through starBase and Protein Atlas databases. Interestingly, GOLM1 knockdown has minimal effect on B7-H3 expression, but reduced B7-H3 expression on tumor cells and soluble B7-H3 level, indicating that GOLM1 is associated with B7-H3 posttranscriptional regulation. Previous study has demonstrated that GOLM1 serves as a specific cargo adaptor to assist with EGFR anchoring on the trans-Golgi network, which helps EGFR recycling back to plasma membrane for persistent function [14]. Here, our results indicated that GOLM1 may serve as the adaptor of B7-H3 for membrane transportation. In addition, high levels of soluble GOLM1 are detected in the serum of several cancers, while B7-H3 protein has also been found in the secretome, including exosomes and other extracellular vesicles [26]. Liang et al. also demonstrated that the exosomes derived from ovarian cancer cell lines OVCAR-3 and IGROV1 both contain GOLM1 and B7-H3 proteins [27], indicating that GOLM1 may also assist with B7-H3 secretion in an exosome-dependent manner. Here, we also confirmed that GOLM1 can physically interact with B7-H3 protein, indicating that GOLM1 may serve as a modulator of B7-H3 protein stability.

Despite its immunosuppressive role in tumor environment, soluble B7-H3 can induce VEGF expression in tumor cells to promote invasion and metastasis [28, 29]. Here, we demonstrated that B7-H3 expression is also involved in ovarian tumor invasion and metastasis in vitro through upregulation of VEGF and MMP-9. GOLM1 knockdown reduced invasion and metastasis through reducing soluble B7-H3, while additional B7-H3 could rescue this phenotype. In addition, GOLM1 knockdown significantly reduced tumor formation and metastasis in immunodeficient mice, which is similar with B7-H3 knockdown group.

In summary, we have first explained that GOLM1 has the correlative expression pattern with B7-H3 in ovarian cancer. GOLM1 regulates membrane B7-H3 expression and B7-H3 secretion but does not regulate B7-H3 mRNA synthesis. Importantly, GOLM1 acts as a vital oncogene by promoting ovarian cancer metastasis through modulating B7-H3 protein maturation and secretion.

## Data Availability

The research data used to support the findings of this study are available from the corresponding author upon request.
